# Utilizing the en face view for unfavorable coronary access after transcatheter aortic valve replacement

**DOI:** 10.1007/s12928-022-00895-7

**Published:** 2022-10-27

**Authors:** Suguru Hirose, Masaki Miyasaka, Norio Tada

**Affiliations:** 1 Department of Cardiology, Sendai Kousei Hospital, 4-15 Hirosemachi, Aoba, Sendai, Miyagi 980-0873 Japan; 2 Department of Cardiovascular Medicine, School of Medicine, Dokkyo Medical University, Tochigi, Japan; 3 Department of Laboratory Medicine, Jikei University School of Medicine, Tokyo, Japan

An 88-year-old woman undergoing transcatheter aortic valve replacement (TAVR) using a 29-mm Evolut PRO+ (Medtronic, Minneapolis, MN, USA) (Fig. 1A) complained of chest pain. We suspected coronary artery disease and consequently planned a coronary angiography (CAG). Computed tomography revealed that the transcatheter heart valve (THV) commissure overlapped the left main coronary artery ostia, which was an unfavorable coronary access case because a catheter cannot cross the cells at the commissures of THV (Fig. 1B–D). We attempted a CAG using bi-plane fluoroscopic system of an en face (right anterior oblique, 63°; cranial [CRA], 49°) and perpendicular view (left anterior oblique, 50°; CRA, 20°) to show the short- and long-axis image of the THV, respectively. Using the en face view made it possible to cross a 5-Fr Judkins left 3.5 catheter through the cell posterior to the C-tab, which is a fluoroscopic marker for one of the THV commissures (Fig. 1E). Following this, we rotated the catheter clockwise anteriorly and were able to achieve selective engagement (Fig. 1F–H).

**Fig. 1:**
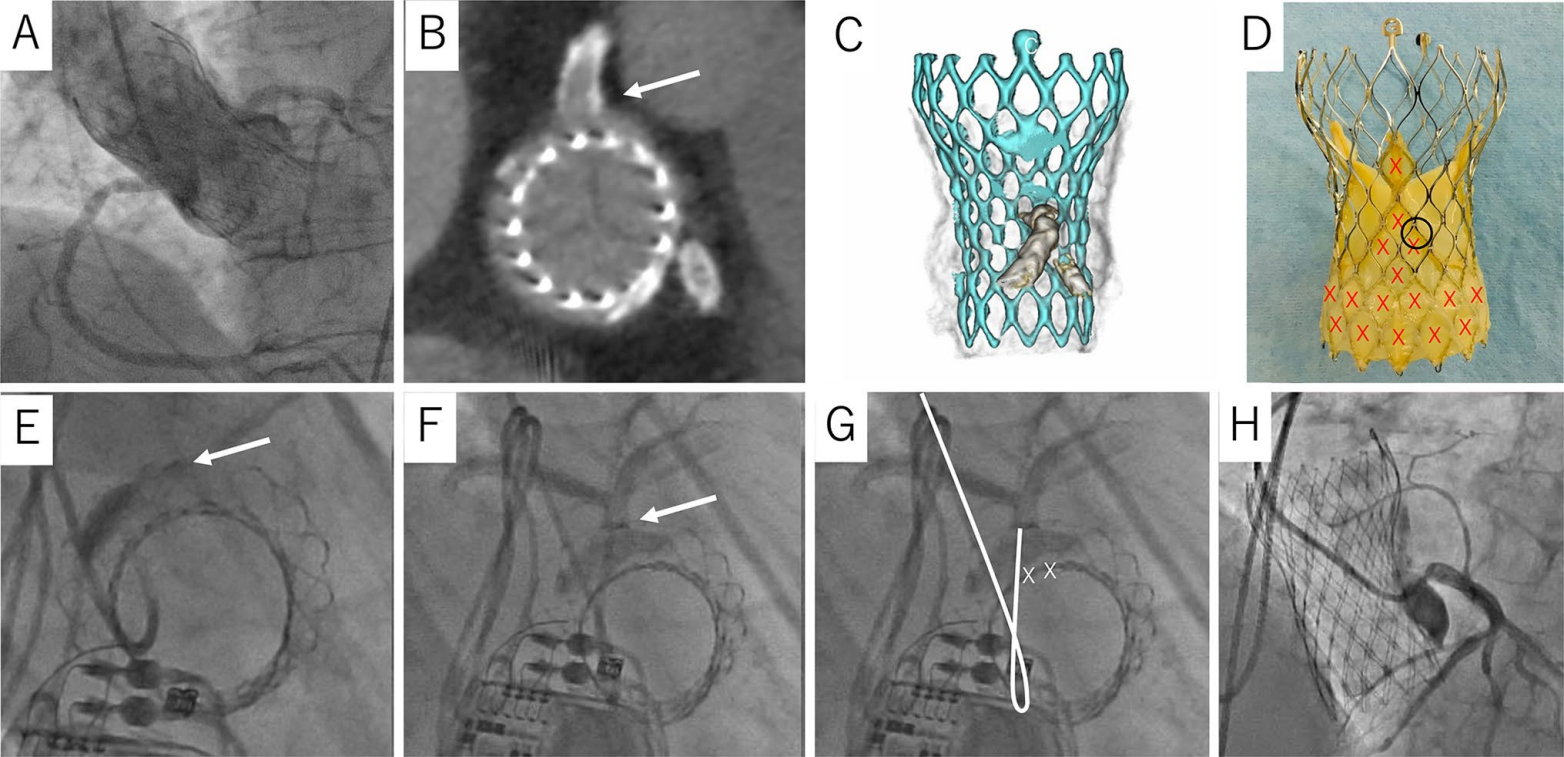
Utilizing the en face view for unfavorable coronary access after TAVR. **A** Aortography after TAVR. **B** The commissure was in front of the left coronary ostia (arrow). **C**, **D** The coronary ostia positioned commissural triangles above the skirt of the Evolut PRO+ (circle). The catheter cannot pass the cell (X mark). **E**, **F** Angiograms taken from the en face views (RAO 63° Cranial 49°). The catheter was passed through the cell posterior to the C-tab (arrow) and rotated clockwise to achieve selective engagement. **G** The same image as **F**. The white line traces the 5-Fr Judkins left 3.5 catheter. Cells cannot pass through (X mark). **H** Selective engagement in the perpendicular view. *TAVR* transcatheter aortic valve replacement, *RAO* right anterior oblique

Ochiai et al. suggested that selective engagement was impossible in all unfavorable coronary access cases with Evolut THVs [1]. We have previously proposed that the en face view is a novel approach; it is useful for understanding the short-axis orientation (anterior–posterior) and rotational manipulation of the catheter in the clockwise/counterclockwise directions [2]. This novel approach was helpful for selective engagement in this case. However, there may still be other difficult cases regarding coronary access, such as high implantation valves, low coronary height, and a shallow sinus of Valsalva. More studies are needed to evaluate the anatomical risk for coronary access and prove the efficacy of a coronary access using the en face view.

## References

[1] Ochiai T, Chakravarty T, Yoon SH (2020). Coronary access after TAVR. J Am Coll Cardiol Interv.

[2] Hirose S, Enta Y, Ishii K (2022). En face view of the transcatheter heart valve from deep right-anterior-oblique cranial position for coronary access after transcatheter aortic valve implantation: a case series. Eur Heart Case Rep.

